# Bis(2,2′:6′,2′′-terpyridine)­ruthenium(II) bis­(perchlorate) hemihydrate

**DOI:** 10.1107/S1600536812043917

**Published:** 2012-10-27

**Authors:** Mariana Kozlowska, Pawel Rodziewicz, Diana Malgorzata Brus, Joanna Breczko, Krzysztof Brzezinski

**Affiliations:** a Institute of Chemistry, University of Bialystok, Hurtowa 1, 15-399 Bialystok, Poland

## Abstract

The asymmetric unit of the title compound, [Ru(C_15_H_11_N_3_)_2_](ClO_4_)_2_·0.5H_2_O, contains one ruthenium–terpiridine complex cation, two perchlorate anions and one half-mol­ecule of water. Face-to-face and face-to-edge π-stacking inter­actions between terpyridine units [centroid–centroid distances = 3.793 (2) and 3.801 (2)  Å] stabilize the crystal lattice The partially occupied water mol­ecule inter­acts with two perchlorate ions *via* O—H⋯O hydrogen bonds. In the crystal lattice, the complex cations, perchlorate ion-water pairs and the second perchlorate anions are arranged into columns along *b* direction.

## Related literature

For the preparation of terpyridine complexes with transition metals, see: Burstall & Nyholm (1952 ▶). For the structures of salts of complexes of ruthenium with terpyridine, see: Craig *et al.* (1998 ▶); Lashgari *et al.* (1999 ▶); Pyo *et al.* (1999 ▶); Tovee *et al.* (2009 ▶); Walstrom *et al.* (2009 ▶). For background to the properties and applications of terpiridine complexes, see: Anders & Schubert (2004 ▶); Constable (2007 ▶); Plonska *et al.* (2002 ▶); Winkler *et al.* (2003 ▶, 2006 ▶).
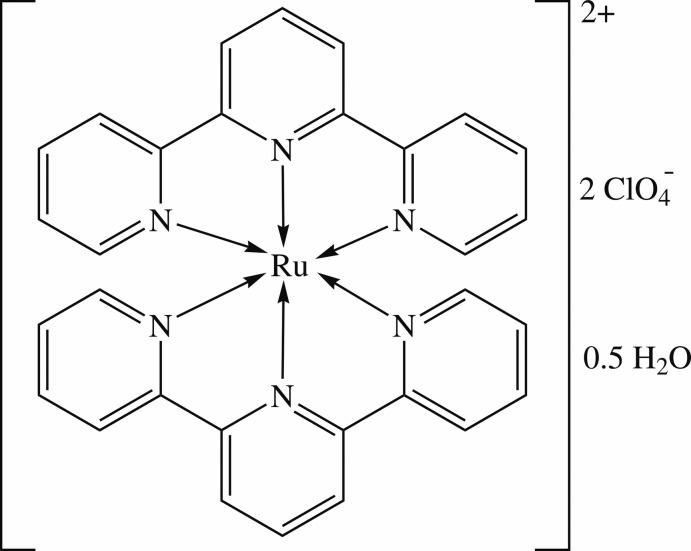


## Experimental

### 

#### Crystal data

[Ru(C_15_H_11_N_3_)_2_](ClO_4_)_2_·0.5H_2_O
*M* *_r_* = 775.51
Monoclinic, 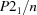

*a* = 8.7676 (2) Å
*b* = 8.8221 (9) Å
*c* = 39.118 (4) Å
β = 93.582 (5)°
*V* = 3019.8 (4) Å^3^
*Z* = 4
Mo *K*α radiation
μ = 0.76 mm^−1^
*T* = 100 K
0.15 × 0.12 × 0.03 mm

#### Data collection

Agilent SuperNova (Dual, Cu at zero, Atlas) diffractometer
Absorption correction: multi-scan (*CrysAlis PRO*; Agilent, 2011 ▶) *T*_min_ = 0.801, *T*_max_ = 1.000
16537 measured reflections
6158 independent reflections
5858 reflections with *I* > 2σ(*I*)
*R*_int_ = 0.027

#### Refinement

*R*[*F* ^2^ > 2σ(*F* ^2^)] = 0.042
*wR*(*F* ^2^) = 0.083
*S* = 1.27
6158 reflections
439 parameters
3 restraints
H atoms treated by a mixture of independent and constrained refinement
Δρ_max_ = 0.65 e Å^−3^
Δρ_min_ = −1.17 e Å^−3^

### 

Data collection: *CrysAlis PRO* (Agilent, 2011 ▶); cell refinement: *CrysAlis PRO*; data reduction: *CrysAlis PRO*; program(s) used to solve structure: *SHELXD* (Sheldrick, 2008 ▶); program(s) used to refine structure: *SHELXL97* (Sheldrick, 2008 ▶); molecular graphics: *ORTEP-3* (Farrugia, 1997 ▶) and *Mercury* (Macrae *et al.*, 2006 ▶); software used to prepare material for publication: *SHELXL97*.

## Supplementary Material

Crystal structure: contains datablock(s) global, I. DOI: 10.1107/S1600536812043917/bt6849sup1.cif

Structure factors: contains datablock(s) I. DOI: 10.1107/S1600536812043917/bt6849Isup2.hkl

Additional supplementary materials:  crystallographic information; 3D view; checkCIF report

## Figures and Tables

**Table 1:** Hydrogen-bond geometry (Å, °) *Cg*1 and *Cg*2 are the centoids of the N3*A*–C15*A* and N3*B*–C15*B* rings, respectively.

*D*—H⋯*A*	*D*—H	H⋯*A*	*D*⋯*A*	*D*—H⋯*A*
O5—H5*A*⋯O2*A* ^i^	0.85 (2)	1.98 (3)	2.790 (6)	159 (7)
O5—H5*B*⋯O1*A*	0.85 (2)	2.03 (3)	2.824 (6)	157 (7)
C2*B*—H2*B*⋯*Cg*1^ii^	0.95	3.09 (1)	3.945 (4)	45 (1)
C14*A*—H14*A*⋯*Cg*2^iii^	0.95	3.01 (1)	3.878 (4)	43 (1)

Symmetry codes: (i) 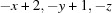; (ii) 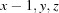; (iii) 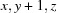.

**Fig. 1.**
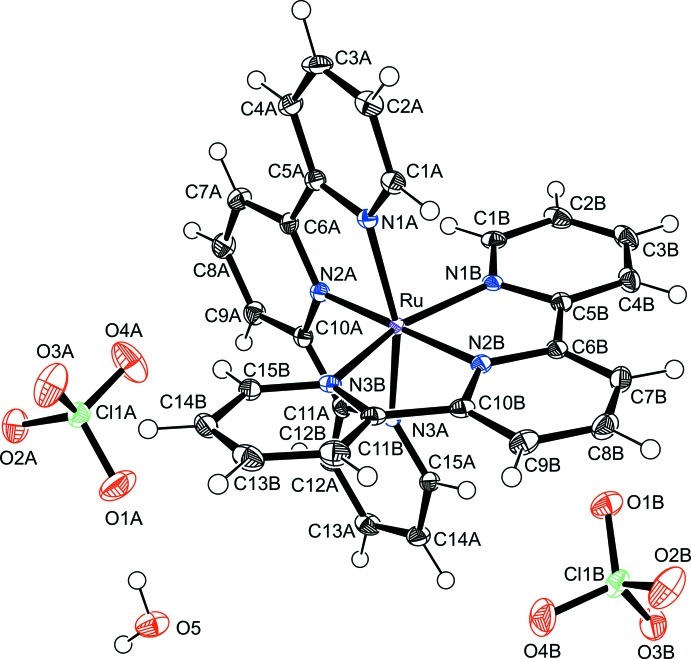
The molecular structure of the compound. Displacement ellipsoids are drawn at the 50% probability level.

**Fig. 2.**
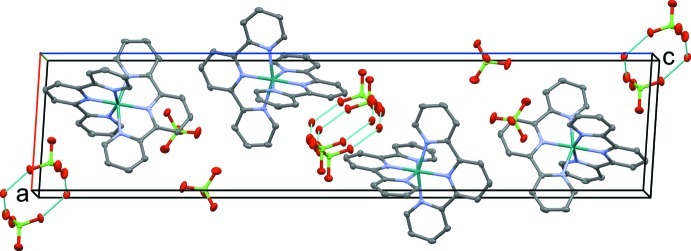
Crystal packing viewed along b direction. Dashed lines represent hydrogen bonds between half-molecule of water and perchlorate anions.

## supplementary crystallographic information

### Comment

A 2,2':6',2''-terpyridine (tpy) compound, as chelating *N*-donor, forms complexes with most of transition metals (Burstall *et al.*, 1952). The polyimine complexes of divalent transition metal cations are well known due to their photophysical and electrochemical properties (Anders *et al.*, 2004; Plonska *et al.*, 2002; Winkler *et al.*, 2003; Winkler *et al.*, 2006). Ruthenium complexes with terpyridyl or bipyridyl ligands might catalyze photochemical water oxidation (Constable, 2007). The metal-to-ligand charge transfer processes in the visible region enable photo- and electroluminescence phenomena and make these applicable in a supramolecular chemistry (Constable, 2007).

The asymmetric unit contains one divalent cation of the ruthenium-terpiridine complex, two perchlorate anions and a water molecule with a half-occupancy (Fig. 1). The crystal lattice is stabilized by terpyridine moieties and respective face-to-face and face-to-edge π-stacking interactions. The partially occupied water molecule and one perchlorate anion are located in a proximity of the inversion center and a symmetry related water-anion pair is generated. Two hydrogen bonds O5—H5A···O1A and H5—H5B···O2A (equivalent anion -*x* + 2,-*y* + 1,*-z*) are formed between water molecule and oxygen atoms of perchlorate units. Geometrical parameters of hydrogen bond interactions are summarized in Table 1. In the crystal lattice each water molecule serves as a bridge between two symmetry dependent perchlorate units (Fig. 2). It is of note, that only one perchlorate unit and its symmetry-mates form hydrogen bonds with water molecules, whereas the second anion interacts with C-bonded hydrogen atoms (Fig 2).

### Experimental

The transition metal complex salt, [Ru^II^(tpy)_2_](ClO_4_)_2_ was prepared according to the procedure described by Burstall *et al.* (1952). Crystals suitable for X-ray diffraction study were obtained at room temperature by a slow evaporation of [Ru^II^(tpy)_2_](ClO_4_)_2_ solution in acetonitrile.

### Refinement

During the initial refinement steps, the occupancy factor for the water molecule was refined and it was in a range of 0.49–0.52. For the final refinement cycles, this occupancy was fixed at 0.5 with isotropic atomic displacement parameters for hydrogen atoms. All H atoms were located in electron density difference maps. C-bonded hydrogen atoms were constrained to idealized positions with C—H distances fixed at 0.95 Å and 1.2*U*_eq_(C). O—H distances were fixed at 0.85 Å with *U*_iso_(H) = 1.5*U*_eq_(C) and the positions of water hydrogen atoms were refined.

### Crystal data

**Table:**

[Ru(C_15_H_11_N_3_)_2_](ClO_4_)_2_·0.5H_2_O	*F*(000) = 1564
*M**_r_* = 775.51	*D*_x_ = 1.706 Mg m^−^^3^
Monoclinic, *P*2_1_/*n*	Mo *K*α radiation, λ = 0.71073 Å
Hall symbol: -P 2yn	Cell parameters from 7538 reflections
*a* = 8.7676 (2) Å	θ = 2.5–26.3°
*b* = 8.8221 (9) Å	µ = 0.76 mm^−^^1^
*c* = 39.118 (4) Å	*T* = 100 K
β = 93.582 (5)°	Plate, red
*V* = 3019.8 (4) Å^3^	0.15 × 0.12 × 0.03 mm
*Z* = 4	

### Data collection

**Table:**

Agilent SuperNova (Dual, Cu at zero, Atlas) diffractometer	6158 independent reflections
Radiation source: SuperNova (Mo) X-ray Source	5858 reflections with *I* > 2σ(*I*)
Mirror monochromator	*R*_int_ = 0.027
Detector resolution: 10.4052 pixels mm^-1^	θ_max_ = 26.4°, θ_min_ = 2.5°
ω scans	*h* = −10→10
Absorption correction: multi-scan (*CrysAlis PRO*; Agilent, 2011)	*k* = 0→11
*T*_min_ = 0.801, *T*_max_ = 1.000	*l* = 0→48
16537 measured reflections	

### Refinement

**Table:**

Refinement on *F*^2^	Primary atom site location: structure-invariant direct methods
Least-squares matrix: full	Secondary atom site location: difference Fourier map
*R*[*F*^2^ > 2σ(*F*^2^)] = 0.042	Hydrogen site location: inferred from neighbouring sites
*wR*(*F*^2^) = 0.083	H atoms treated by a mixture of independent and constrained refinement
*S* = 1.27	*w* = 1/[σ^2^(*F*_o_^2^) + (0.0111*P*)^2^ + 7.0042*P*] where *P* = (*F*_o_^2^ + 2*F*_c_^2^)/3
6158 reflections	(Δ/σ)_max_ = 0.001
439 parameters	Δρ_max_ = 0.65 e Å^−^^3^
3 restraints	Δρ_min_ = −1.17 e Å^−^^3^

### Special details

**Table:**

Geometry. All e.s.d.'s (except the e.s.d. in the dihedral angle between two l.s. planes) are estimated using the full covariance matrix. The cell e.s.d.'s are taken into account individually in the estimation of e.s.d.'s in distances, angles and torsion angles; correlations between e.s.d.'s in cell parameters are only used when they are defined by crystal symmetry. An approximate (isotropic) treatment of cell e.s.d.'s is used for estimating e.s.d.'s involving l.s. planes.
Refinement. Refinement of *F*^2^ against ALL reflections. The weighted *R*-factor *wR* and goodness of fit *S* are based on *F*^2^, conventional *R*-factors *R* are based on *F*, with *F* set to zero for negative *F*^2^. The threshold expression of *F*^2^ > σ(*F*^2^) is used only for calculating *R*-factors(gt) *etc*. and is not relevant to the choice of reflections for refinement. *R*-factors based on *F*^2^ are statistically about twice as large as those based on *F*, and *R*- factors based on ALL data will be even larger.

### Fractional atomic coordinates and isotropic or equivalent isotropic displacement parameters (Å^2^)

**Table:**

	*x*	*y*	*z*	*U*_iso_*/*U*_eq_	Occ. (<1)
Ru1	0.35193 (3)	0.33315 (3)	0.127794 (6)	0.01027 (7)	
N1A	0.2631 (3)	0.1164 (3)	0.12359 (7)	0.0121 (5)	
N3A	0.4324 (3)	0.5408 (3)	0.11162 (7)	0.0122 (5)	
C15A	0.4968 (3)	0.6520 (4)	0.13099 (8)	0.0142 (6)	
H15A	0.5039	0.6399	0.1552	0.017*	
C1A	0.2479 (3)	0.0135 (4)	0.14867 (8)	0.0155 (7)	
H1A	0.2837	0.0386	0.1714	0.019*	
C10A	0.3495 (3)	0.4333 (4)	0.05723 (8)	0.0139 (6)	
C10B	0.5227 (3)	0.3134 (4)	0.19339 (8)	0.0145 (6)	
C11A	0.4207 (3)	0.5607 (4)	0.07649 (8)	0.0145 (6)	
C5A	0.2170 (3)	0.0758 (4)	0.09058 (8)	0.0142 (6)	
C12A	0.4743 (4)	0.6915 (4)	0.06155 (9)	0.0179 (7)	
H12A	0.4635	0.7042	0.0374	0.022*	
C2A	0.1819 (4)	−0.1275 (4)	0.14247 (9)	0.0181 (7)	
H2A	0.1724	−0.1975	0.1607	0.022*	
C15B	0.6578 (3)	0.1840 (4)	0.11219 (8)	0.0156 (7)	
H15B	0.6228	0.1879	0.0887	0.019*	
C4A	0.1491 (4)	−0.0635 (4)	0.08320 (9)	0.0174 (7)	
H4A	0.1162	−0.0888	0.0603	0.021*	
C12B	0.7609 (4)	0.1731 (4)	0.17951 (9)	0.0199 (7)	
H12B	0.7949	0.1700	0.2030	0.024*	
C8A	0.2508 (4)	0.2943 (4)	0.00816 (8)	0.0213 (7)	
H8A	0.2296	0.2866	−0.0159	0.026*	
C9A	0.3187 (4)	0.4247 (4)	0.02177 (8)	0.0184 (7)	
H9A	0.3437	0.5064	0.0073	0.022*	
C14A	0.5534 (4)	0.7834 (4)	0.11718 (9)	0.0189 (7)	
H14A	0.5985	0.8594	0.1317	0.023*	
C3A	0.1300 (4)	−0.1650 (4)	0.10941 (9)	0.0211 (7)	
H3A	0.0816	−0.2599	0.1048	0.025*	
C13A	0.5437 (4)	0.8035 (4)	0.08197 (9)	0.0205 (7)	
H13A	0.5838	0.8921	0.0720	0.025*	
C4B	0.0091 (4)	0.5255 (4)	0.18713 (9)	0.0193 (7)	
H4B	0.0076	0.5521	0.2106	0.023*	
C7B	0.3040 (4)	0.4475 (4)	0.23114 (8)	0.0196 (7)	
H7B	0.2281	0.4937	0.2440	0.024*	
N2B	0.3874 (3)	0.3573 (3)	0.17813 (6)	0.0124 (5)	
C6B	0.2779 (3)	0.4223 (4)	0.19610 (8)	0.0143 (6)	
C14B	0.7970 (4)	0.1152 (4)	0.12103 (9)	0.0184 (7)	
H14B	0.8565	0.0731	0.1039	0.022*	
C1B	0.0186 (4)	0.4455 (4)	0.11975 (9)	0.0163 (7)	
H1B	0.0203	0.4168	0.0964	0.020*	
C13B	0.8476 (4)	0.1089 (4)	0.15514 (9)	0.0216 (7)	
H13B	0.9417	0.0606	0.1618	0.026*	
C5B	0.1388 (4)	0.4593 (4)	0.17462 (8)	0.0149 (7)	
C2B	−0.1131 (4)	0.5101 (4)	0.13105 (9)	0.0208 (7)	
H2B	−0.1997	0.5253	0.1156	0.025*	
C3B	−0.1173 (4)	0.5522 (4)	0.16509 (9)	0.0223 (8)	
H3B	−0.2059	0.5988	0.1732	0.027*	
C8B	0.4433 (4)	0.4036 (4)	0.24683 (9)	0.0215 (7)	
H8B	0.4625	0.4199	0.2707	0.026*	
C9B	0.5547 (4)	0.3367 (4)	0.22841 (8)	0.0192 (7)	
H9B	0.6501	0.3074	0.2393	0.023*	
N1B	0.1441 (3)	0.4219 (3)	0.14059 (7)	0.0127 (5)	
N2A	0.3132 (3)	0.3157 (3)	0.07744 (6)	0.0126 (5)	
C6A	0.2464 (3)	0.1891 (4)	0.06429 (8)	0.0142 (6)	
N3B	0.5705 (3)	0.2454 (3)	0.13564 (6)	0.0123 (5)	
C7A	0.2136 (4)	0.1753 (4)	0.02910 (8)	0.0198 (7)	
H7A	0.1668	0.0862	0.0197	0.024*	
C11B	0.6234 (3)	0.2424 (4)	0.16936 (8)	0.0146 (7)	
Cl1A	0.78661 (9)	0.20109 (9)	0.02625 (2)	0.01935 (18)	
O2A	0.8387 (3)	0.2126 (3)	−0.00779 (6)	0.0306 (6)	
O4A	0.6256 (3)	0.2268 (4)	0.02484 (7)	0.0426 (8)	
O3A	0.8209 (4)	0.0534 (3)	0.03981 (7)	0.0383 (7)	
O1A	0.8611 (4)	0.3124 (3)	0.04797 (7)	0.0407 (7)	
Cl1B	0.52415 (9)	0.83287 (10)	0.22156 (2)	0.02087 (18)	
O2B	0.5394 (3)	0.7741 (3)	0.25576 (7)	0.0369 (7)	
O1B	0.3911 (3)	0.7690 (3)	0.20366 (6)	0.0279 (6)	
O3B	0.5070 (3)	0.9946 (3)	0.22300 (7)	0.0297 (6)	
O4B	0.6574 (3)	0.7965 (4)	0.20354 (7)	0.0433 (8)	
O5	0.9985 (6)	0.6020 (6)	0.05038 (12)	0.0266 (11)	0.50
H5A	1.044 (8)	0.639 (8)	0.0339 (13)	0.040*	0.50
H5B	0.950 (8)	0.523 (6)	0.0438 (18)	0.040*	0.50

### Atomic displacement parameters (Å^2^)

**Table:**

	*U*^11^	*U*^22^	*U*^33^	*U*^12^	*U*^13^	*U*^23^
Ru1	0.00989 (12)	0.00929 (12)	0.01172 (13)	−0.00013 (10)	0.00132 (9)	−0.00072 (10)
N1A	0.0079 (12)	0.0103 (13)	0.0179 (13)	0.0022 (10)	0.0002 (10)	−0.0004 (11)
N3A	0.0100 (12)	0.0096 (13)	0.0173 (13)	0.0017 (10)	0.0025 (10)	−0.0008 (11)
C15A	0.0119 (14)	0.0129 (16)	0.0179 (16)	0.0005 (12)	0.0008 (12)	−0.0028 (13)
C1A	0.0130 (15)	0.0161 (17)	0.0171 (16)	0.0008 (13)	0.0000 (12)	0.0015 (13)
C10A	0.0114 (15)	0.0123 (16)	0.0181 (16)	0.0039 (12)	0.0010 (12)	0.0001 (13)
C10B	0.0158 (15)	0.0109 (16)	0.0168 (16)	−0.0039 (12)	0.0002 (12)	0.0007 (13)
C11A	0.0123 (15)	0.0145 (16)	0.0171 (16)	0.0030 (13)	0.0031 (12)	−0.0005 (13)
C5A	0.0101 (15)	0.0129 (16)	0.0194 (16)	0.0024 (12)	0.0003 (12)	−0.0015 (13)
C12A	0.0190 (16)	0.0153 (17)	0.0198 (17)	−0.0005 (13)	0.0031 (13)	0.0031 (14)
C2A	0.0150 (16)	0.0119 (16)	0.0273 (18)	0.0037 (13)	0.0013 (13)	0.0053 (14)
C15B	0.0171 (16)	0.0126 (16)	0.0175 (16)	−0.0028 (13)	0.0047 (12)	−0.0005 (13)
C4A	0.0153 (16)	0.0145 (17)	0.0221 (17)	0.0000 (13)	−0.0012 (13)	−0.0031 (14)
C12B	0.0168 (16)	0.0195 (17)	0.0228 (17)	−0.0005 (14)	−0.0030 (13)	0.0026 (15)
C8A	0.0236 (18)	0.028 (2)	0.0122 (16)	−0.0017 (15)	0.0000 (13)	−0.0018 (14)
C9A	0.0204 (17)	0.0187 (18)	0.0162 (16)	0.0000 (14)	0.0026 (13)	0.0009 (14)
C14A	0.0165 (16)	0.0143 (16)	0.0260 (18)	−0.0020 (13)	0.0018 (13)	−0.0038 (14)
C3A	0.0157 (16)	0.0118 (16)	0.036 (2)	−0.0042 (14)	0.0000 (14)	−0.0010 (15)
C13A	0.0193 (17)	0.0129 (17)	0.0297 (19)	−0.0023 (13)	0.0047 (14)	0.0044 (14)
C4B	0.0207 (17)	0.0141 (17)	0.0240 (18)	0.0010 (14)	0.0093 (14)	0.0003 (14)
C7B	0.0258 (18)	0.0160 (17)	0.0178 (17)	−0.0032 (14)	0.0088 (13)	−0.0030 (14)
N2B	0.0133 (13)	0.0081 (13)	0.0161 (13)	−0.0015 (10)	0.0028 (10)	−0.0007 (10)
C6B	0.0149 (15)	0.0089 (15)	0.0195 (16)	−0.0024 (12)	0.0044 (12)	−0.0017 (13)
C14B	0.0152 (16)	0.0146 (16)	0.0262 (18)	0.0000 (13)	0.0073 (13)	−0.0003 (14)
C1B	0.0159 (16)	0.0100 (16)	0.0228 (17)	−0.0028 (13)	0.0005 (13)	0.0017 (13)
C13B	0.0147 (16)	0.0184 (18)	0.032 (2)	0.0010 (14)	0.0013 (14)	0.0041 (15)
C5B	0.0184 (16)	0.0076 (15)	0.0194 (16)	−0.0023 (12)	0.0065 (13)	0.0006 (13)
C2B	0.0134 (16)	0.0159 (17)	0.033 (2)	0.0005 (13)	0.0019 (14)	0.0046 (15)
C3B	0.0167 (17)	0.0157 (17)	0.036 (2)	0.0030 (14)	0.0111 (14)	0.0021 (15)
C8B	0.0292 (19)	0.0219 (19)	0.0134 (16)	−0.0064 (15)	0.0008 (13)	−0.0018 (14)
C9B	0.0212 (17)	0.0187 (17)	0.0171 (16)	−0.0012 (14)	−0.0049 (13)	0.0000 (14)
N1B	0.0122 (13)	0.0083 (13)	0.0179 (14)	−0.0028 (10)	0.0030 (10)	−0.0004 (11)
N2A	0.0111 (12)	0.0111 (13)	0.0155 (13)	0.0013 (10)	0.0012 (10)	0.0000 (11)
C6A	0.0116 (14)	0.0127 (16)	0.0185 (16)	0.0030 (12)	0.0017 (12)	−0.0027 (13)
N3B	0.0111 (12)	0.0101 (13)	0.0155 (13)	−0.0026 (10)	0.0000 (10)	0.0010 (11)
C7A	0.0198 (16)	0.0204 (18)	0.0190 (17)	−0.0021 (15)	−0.0010 (13)	−0.0044 (14)
C11B	0.0144 (15)	0.0120 (16)	0.0175 (16)	−0.0049 (13)	0.0008 (12)	0.0004 (13)
Cl1A	0.0207 (4)	0.0201 (4)	0.0172 (4)	0.0005 (3)	0.0013 (3)	−0.0022 (3)
O2A	0.0369 (15)	0.0372 (16)	0.0184 (13)	0.0012 (13)	0.0073 (11)	−0.0027 (12)
O4A	0.0226 (14)	0.072 (2)	0.0337 (16)	0.0097 (15)	0.0027 (12)	0.0139 (16)
O3A	0.062 (2)	0.0264 (15)	0.0263 (15)	0.0128 (14)	−0.0007 (13)	0.0011 (12)
O1A	0.0560 (19)	0.0395 (18)	0.0263 (15)	−0.0183 (15)	0.0012 (13)	−0.0127 (13)
Cl1B	0.0236 (4)	0.0194 (4)	0.0191 (4)	0.0054 (3)	−0.0023 (3)	−0.0056 (3)
O2B	0.0604 (19)	0.0273 (15)	0.0211 (14)	0.0101 (14)	−0.0127 (13)	0.0005 (12)
O1B	0.0301 (14)	0.0306 (15)	0.0218 (13)	−0.0036 (12)	−0.0073 (11)	−0.0022 (11)
O3B	0.0389 (16)	0.0187 (13)	0.0330 (15)	−0.0019 (12)	0.0143 (12)	−0.0010 (12)
O4B	0.0276 (15)	0.059 (2)	0.0432 (17)	0.0136 (14)	0.0025 (13)	−0.0240 (16)
O5	0.034 (3)	0.025 (3)	0.021 (3)	−0.010 (2)	−0.002 (2)	0.005 (2)

### Geometric parameters (Å, º)

**Table:**

Ru1—N2A	1.984 (3)	C14A—C13A	1.386 (5)
Ru1—N2B	1.986 (3)	C14A—H14A	0.9500
Ru1—N1A	2.067 (3)	C3A—H3A	0.9500
Ru1—N3B	2.072 (3)	C13A—H13A	0.9500
Ru1—N1B	2.073 (3)	C4B—C3B	1.381 (5)
Ru1—N3A	2.076 (3)	C4B—C5B	1.394 (4)
N1A—C1A	1.350 (4)	C4B—H4B	0.9500
N1A—C5A	1.376 (4)	C7B—C8B	1.388 (5)
N3A—C15A	1.342 (4)	C7B—C6B	1.393 (4)
N3A—C11A	1.383 (4)	C7B—H7B	0.9500
C15A—C14A	1.384 (5)	N2B—C6B	1.352 (4)
C15A—H15A	0.9500	C6B—C5B	1.474 (4)
C1A—C2A	1.387 (5)	C14B—C13B	1.381 (5)
C1A—H1A	0.9500	C14B—H14B	0.9500
C10A—N2A	1.354 (4)	C1B—N1B	1.345 (4)
C10A—C9A	1.399 (4)	C1B—C2B	1.384 (5)
C10A—C11A	1.470 (4)	C1B—H1B	0.9500
C10B—N2B	1.351 (4)	C13B—H13B	0.9500
C10B—C9B	1.396 (4)	C5B—N1B	1.375 (4)
C10B—C11B	1.469 (4)	C2B—C3B	1.385 (5)
C11A—C12A	1.389 (4)	C2B—H2B	0.9500
C5A—C4A	1.388 (4)	C3B—H3B	0.9500
C5A—C6A	1.468 (4)	C8B—C9B	1.382 (5)
C12A—C13A	1.387 (5)	C8B—H8B	0.9500
C12A—H12A	0.9500	C9B—H9B	0.9500
C2A—C3A	1.384 (5)	N2A—C6A	1.349 (4)
C2A—H2A	0.9500	C6A—C7A	1.394 (4)
C15B—N3B	1.344 (4)	N3B—C11B	1.371 (4)
C15B—C14B	1.388 (4)	C7A—H7A	0.9500
C15B—H15B	0.9500	Cl1A—O4A	1.427 (3)
C4A—C3A	1.380 (5)	Cl1A—O1A	1.430 (3)
C4A—H4A	0.9500	Cl1A—O3A	1.432 (3)
C12B—C13B	1.378 (5)	Cl1A—O2A	1.438 (3)
C12B—C11B	1.387 (4)	Cl1B—O2B	1.433 (3)
C12B—H12B	0.9500	Cl1B—O3B	1.437 (3)
C8A—C7A	1.384 (5)	Cl1B—O1B	1.437 (3)
C8A—C9A	1.386 (5)	Cl1B—O4B	1.438 (3)
C8A—H8A	0.9500	O5—H5A	0.85 (2)
C9A—H9A	0.9500	O5—H5B	0.85 (2)
			
N2A—Ru1—N2B	178.09 (11)	C12A—C13A—H13A	120.7
N2A—Ru1—N1A	78.99 (10)	C3B—C4B—C5B	119.5 (3)
N2B—Ru1—N1A	102.30 (10)	C3B—C4B—H4B	120.2
N2A—Ru1—N3B	102.56 (10)	C5B—C4B—H4B	120.2
N2B—Ru1—N3B	78.88 (10)	C8B—C7B—C6B	118.4 (3)
N1A—Ru1—N3B	90.37 (10)	C8B—C7B—H7B	120.8
N2A—Ru1—N1B	99.82 (10)	C6B—C7B—H7B	120.8
N2B—Ru1—N1B	78.78 (10)	C6B—N2B—C10B	121.6 (3)
N1A—Ru1—N1B	92.06 (10)	C6B—N2B—Ru1	119.3 (2)
N3B—Ru1—N1B	157.54 (10)	C10B—N2B—Ru1	119.1 (2)
N2A—Ru1—N3A	78.81 (10)	N2B—C6B—C7B	120.1 (3)
N2B—Ru1—N3A	99.93 (10)	N2B—C6B—C5B	112.7 (3)
N1A—Ru1—N3A	157.73 (10)	C7B—C6B—C5B	127.2 (3)
N3B—Ru1—N3A	92.65 (10)	C13B—C14B—C15B	118.9 (3)
N1B—Ru1—N3A	93.49 (10)	C13B—C14B—H14B	120.5
C1A—N1A—C5A	118.1 (3)	C15B—C14B—H14B	120.5
C1A—N1A—Ru1	128.2 (2)	N1B—C1B—C2B	122.5 (3)
C5A—N1A—Ru1	113.7 (2)	N1B—C1B—H1B	118.7
C15A—N3A—C11A	118.1 (3)	C2B—C1B—H1B	118.7
C15A—N3A—Ru1	127.7 (2)	C12B—C13B—C14B	119.6 (3)
C11A—N3A—Ru1	114.1 (2)	C12B—C13B—H13B	120.2
N3A—C15A—C14A	122.7 (3)	C14B—C13B—H13B	120.2
N3A—C15A—H15A	118.7	N1B—C5B—C4B	121.2 (3)
C14A—C15A—H15A	118.7	N1B—C5B—C6B	114.9 (3)
N1A—C1A—C2A	122.4 (3)	C4B—C5B—C6B	123.8 (3)
N1A—C1A—H1A	118.8	C1B—C2B—C3B	119.4 (3)
C2A—C1A—H1A	118.8	C1B—C2B—H2B	120.3
N2A—C10A—C9A	120.0 (3)	C3B—C2B—H2B	120.3
N2A—C10A—C11A	113.2 (3)	C4B—C3B—C2B	119.1 (3)
C9A—C10A—C11A	126.8 (3)	C4B—C3B—H3B	120.5
N2B—C10B—C9B	120.5 (3)	C2B—C3B—H3B	120.5
N2B—C10B—C11B	112.7 (3)	C9B—C8B—C7B	121.3 (3)
C9B—C10B—C11B	126.8 (3)	C9B—C8B—H8B	119.3
N3A—C11A—C12A	121.1 (3)	C7B—C8B—H8B	119.3
N3A—C11A—C10A	114.5 (3)	C8B—C9B—C10B	118.0 (3)
C12A—C11A—C10A	124.3 (3)	C8B—C9B—H9B	121.0
N1A—C5A—C4A	121.5 (3)	C10B—C9B—H9B	121.0
N1A—C5A—C6A	115.2 (3)	C1B—N1B—C5B	118.3 (3)
C4A—C5A—C6A	123.3 (3)	C1B—N1B—Ru1	127.6 (2)
C13A—C12A—C11A	119.8 (3)	C5B—N1B—Ru1	114.1 (2)
C13A—C12A—H12A	120.1	C6A—N2A—C10A	121.6 (3)
C11A—C12A—H12A	120.1	C6A—N2A—Ru1	119.1 (2)
C3A—C2A—C1A	119.2 (3)	C10A—N2A—Ru1	119.2 (2)
C3A—C2A—H2A	120.4	N2A—C6A—C7A	120.5 (3)
C1A—C2A—H2A	120.4	N2A—C6A—C5A	112.8 (3)
N3B—C15B—C14B	122.4 (3)	C7A—C6A—C5A	126.7 (3)
N3B—C15B—H15B	118.8	C15B—N3B—C11B	118.5 (3)
C14B—C15B—H15B	118.8	C15B—N3B—Ru1	127.6 (2)
C3A—C4A—C5A	119.4 (3)	C11B—N3B—Ru1	113.8 (2)
C3A—C4A—H4A	120.3	C8A—C7A—C6A	118.5 (3)
C5A—C4A—H4A	120.3	C8A—C7A—H7A	120.8
C13B—C12B—C11B	119.4 (3)	C6A—C7A—H7A	120.8
C13B—C12B—H12B	120.3	N3B—C11B—C12B	121.3 (3)
C11B—C12B—H12B	120.3	N3B—C11B—C10B	115.3 (3)
C7A—C8A—C9A	120.9 (3)	C12B—C11B—C10B	123.4 (3)
C7A—C8A—H8A	119.5	O4A—Cl1A—O1A	109.1 (2)
C9A—C8A—H8A	119.5	O4A—Cl1A—O3A	110.07 (19)
C8A—C9A—C10A	118.5 (3)	O1A—Cl1A—O3A	109.10 (18)
C8A—C9A—H9A	120.7	O4A—Cl1A—O2A	108.88 (16)
C10A—C9A—H9A	120.7	O1A—Cl1A—O2A	109.99 (17)
C15A—C14A—C13A	119.6 (3)	O3A—Cl1A—O2A	109.65 (17)
C15A—C14A—H14A	120.2	O2B—Cl1B—O3B	109.08 (16)
C13A—C14A—H14A	120.2	O2B—Cl1B—O1B	109.78 (17)
C4A—C3A—C2A	119.4 (3)	O3B—Cl1B—O1B	108.93 (16)
C4A—C3A—H3A	120.3	O2B—Cl1B—O4B	110.22 (18)
C2A—C3A—H3A	120.3	O3B—Cl1B—O4B	109.33 (18)
C14A—C13A—C12A	118.6 (3)	O1B—Cl1B—O4B	109.47 (16)
C14A—C13A—H13A	120.7	H5A—O5—H5B	110 (5)

### Hydrogen-bond geometry (Å, º)

*Cg*1 and *Cg*2 are the centoids of the N3A–C15A and N3B–C15B rings, respectively.

**Table:**

*D*—H···*A*	*D*—H	H···*A*	*D*···*A*	*D*—H···*A*
O5—H5*A*···O2*A*^i^	0.85 (2)	1.98 (3)	2.790 (6)	159 (7)
O5—H5*B*···O1*A*	0.85 (2)	2.03 (3)	2.824 (6)	157 (7)
C2*B*—H2*B*···*Cg*1^ii^	0.95	3.09 (1)	3.945 (4)	45 (1)
C14*A*—H14*A*···*Cg*2^iii^	0.95	3.01 (1)	3.878 (4)	43 (1)

Symmetry codes: (i) −*x*+2, −*y*+1, −*z*; (ii) *x*−1, *y*, *z*; (iii) *x*, *y*+1, *z*.
